# Promoting physical activity among cancer survivors: an umbrella review of systematic reviews

**DOI:** 10.1007/s00520-023-07760-0

**Published:** 2023-04-25

**Authors:** Lin Qiu, Maodie Ye, Yang Tong, Yongmei Jin

**Affiliations:** 1 Department of Nursing, 7Th Hospital Affiliated to Shanghai University of Traditional Chinese Medicine, Shanghai, China; 2 Department of Nail-Breast Hernia Surgery, 7Th Hospital Affiliated to Shanghai University of Traditional Chinese Medicine, Shanghai, China

**Keywords:** Physical activity, Cancer, Umbrella review, Evidence-based medicine

## Abstract

**Purpose:**

Exercise is the core element of rehabilitation for cancer patients. However, most of the patients’ exercise levels failed to meet the indicators recommended by the guidelines or even decreased. Therefore, this umbrella review aims to provide an overview of review articles addressing the evidence of interventions to promote physical activity behavior change and increase physical activity among cancer patients.

**Methods:**

We searched nine databases from inception to 12 May 2022 to obtain systematic reviews and meta-analyses of interventions to promote physical activity among cancer patients. The AMSTAR-2 was used for the quality assessment.

**Results:**

Twenty-six individual systematic reviews including 13 studies performed meta-analyses. A total of 16 studies’ designs were all in randomized controlled trial. Most reviews included studies that were mainly delivered in home settings. The most frequent and mean duration of the interventions was 12 weeks. Interventions mainly included electronic, wearable health technology-based, behavior change techniques (BCTs), and theory-based strategies.

**Conclusions:**

Electronic, wearable health technology-based, BCTs, and theory-based interventions were effective and feasible in promoting physical activity in cancer survivors. Clinical practitioners should take corresponding intervention measures according to the characteristics of patients in different groups.

**Implications for cancer survivors:**

Future research may benefit cancer survivors by more comprehensively applying electronic, wearable health technology-based, BCTs, and theory-based interventions.

**Supplementary Information:**

The online version contains supplementary material available at 10.1007/s00520-023-07760-0.

## Background

With socio-economic development and the prevalence of various cancer risk factors, the burden of cancer morbidity and mortality worldwide is increasing rapidly [1, 2]. In 2020, there are an estimated 1930 million new cancer cases and nearly 10 million cancer deaths worldwide [1]. Cancer patients have a higher cure rate and survival rate under the popularization of early screening and the progress of medical technology. Exercise is one of the vital measures for rehabilitation of many chronic diseases, improving the quality of life and reducing mortality [3, 4]. At the same time, exercise has a positive impact on anxiety, depression, fatigue, and quality of life decline caused by cancer or treatment [5]. Therefore, multidisciplinary cancer teams should encourage cancer patients to exercise.

Aerobic exercise and resistance exercise are safe, feasible, and effective in cancer patients during and after adjuvant therapy [6, 7]. According to the exercise guide for cancer patients, cancer patients should gradually resume their daily activities after surgery and recommended at least 150 min of aerobic exercise and 2 ~ 3 times of resistance training per week [5]. However, few patients can achieve this index. A 1-year study by Arem et al. [8] found that only 33% of women reached 150 min of exercise per week, suggesting that the physical activity (PA) level of breast cancer patients during treatment was reduced, and they also had less activity in the late stage of treatment. Therefore, after excluding exercise contraindications, when providing exercise advice and intervention for patients, attention should be paid to the maintenance or improvement of exercise compliance of patients to alleviate related symptoms and improve their quality of life of patients.

At present, more systematic evaluations focus on intervention measures for promoting exercise in cancer patients. Behavioral change techniques are commonly used to improve patient compliance. Behavioral change strategies, such as pedometers, motivational interviews, and materials, can improve patient compliance with exercise and increase their exercise level [9]. Lopez et al. [10] reported that high-intensity resistance exercise and low-intensity resistance exercise are equally effective in patients with prostate cancer, and low exercise doses may help to reduce movement disorders and enhance compliance. The purpose of this umbrella review is to systematically summarize the interventions to promote physical activity behavior change and increase physical activity among cancer patients. More specifically, it aims to establish a clear cancer movement promotion plan (i.e., settings, length, measurement tools, and interventions), provide evidence to promote the movement of cancer patients, and can be used for clinical medical staff to develop exercise plans and provide suggestions for patients.

## Methods and analysis

### Protocol registration

This study was conducted according to the Preferred Reporting Items for Systematic Reviews and Meta-Analyses (PRISMA-P) [11] and registered at Prospective Register of Systematic Reviews (PROSPERO) (https://www.crd.york.ac.uk/PROSPERO/; CRD42022316194).

### Literature search strategy

Two authors (QL, YMD) independently and systematically searched the following databases, PubMed, Embase, MEDLINE, Cochrane Library, Scopus, CINAHL, Web of Science, OVID, and Research Square (gray literature) for studies published from inception to 12 May 2022.

The search strategy was developed following the participants, intervention, comparison, and outcomes (PICOs) components. The retrieval was carried out by the combination of keywords and free words (Supplemental File 1). Keywords were adjusted across databases. And we re-runned it before the final analysis (July 18) to ensure that there was no new research.

## Inclusion/exclusion criteria for study selection

The inclusion criteria for this umbrella review according to PICOS format are as follows: (1) types of participants, cancer patients either completed or undergoing treatment; (2) types of interventions, any interventions that may promote or maintain physical activity behavior change and adherence, including but not limited to the motivational strategies, eHealth interventions, and behavior change techniques; (3) types of comparison, compares the intervention to an alternative intervention or usual care; (4) types of outcome, the primary outcome was physical activity behavior (self-reported or objectively measured) and secondary outcomes were including but not limited to physical activity adherence, self-efficacy, physical function, and quality of life; (5) types of studies, systematic reviews and meta-analyses of a quantitative or qualitative study. Criterion (5), systematic reviews that include qualitative and quantitative studies, aims to enrich the evidence.

Studies were excluded if they (1) reported the efficacy of a physical activity intervention that did not involve physical activity level or adherence or (2) were available as a conference abstract only.

## Selection of studies and data extraction

Importing all search results into EndNote X9, title and abstract screening were conducted concurrently by two investigators trained in evidence-based courses (e.g., training in theories, principles, and methods of evidence-based medicine). If the title and abstract lack sufficient information to make a decision, the article was carried forward to the full-text screening stage. Full texts were obtained and screened independently by two investigators according to the inclusion and exclusion criteria of the literature.

The included literature was extracted after the literature quality evaluation by the self-designed standardized table to extract data; the main extracted content are as follows: title, first author, publication year, objective, study designs, number of studies included, analysis method, quality assessment tools, population, intervention, outcome measure, etc. Two researchers did data extraction independently, and inconsistencies were resolved by discussion.

## Methodological quality assessment

The methodological quality of the included systematic reviews and meta-analyses was assessed using AMSTAR-2 [12], which includes 16 items. According to the instrument, two reviewers classified the results of included systematic reviews as high, moderate, low, and critically low. If the study has no or one non-critical weakness, we appraised it as high; if more than one non-critical weakness, we appraised it as moderate; if one critical flaw with or without non-critical weakness, we appraised it as low; and if more than one critical flaw with or without non-critical weakness, we appraised it as critically low.

Our study was founded on the concept of evidence from multiple sources. Included studies had interventional and qualitative studies. At the same time, the interventions and outcome indicators of this study are diverse, and the Grading of Recommendations Assessment, Development and Evaluation (GRADE) is not applicable. Therefore, the evidence was pre-ranking using the Joanna Briggs Institute evidence pre-ranking system [13]. According to the type of included studies, it is divided into five levels from level 1 to 5.

## Data analysis

Due to the factors such as different intervention techniques, different application times, and different research objects, this study only conducts a descriptive analysis of the included studies.

## Results

### Literature selection

**Fig. 1:**
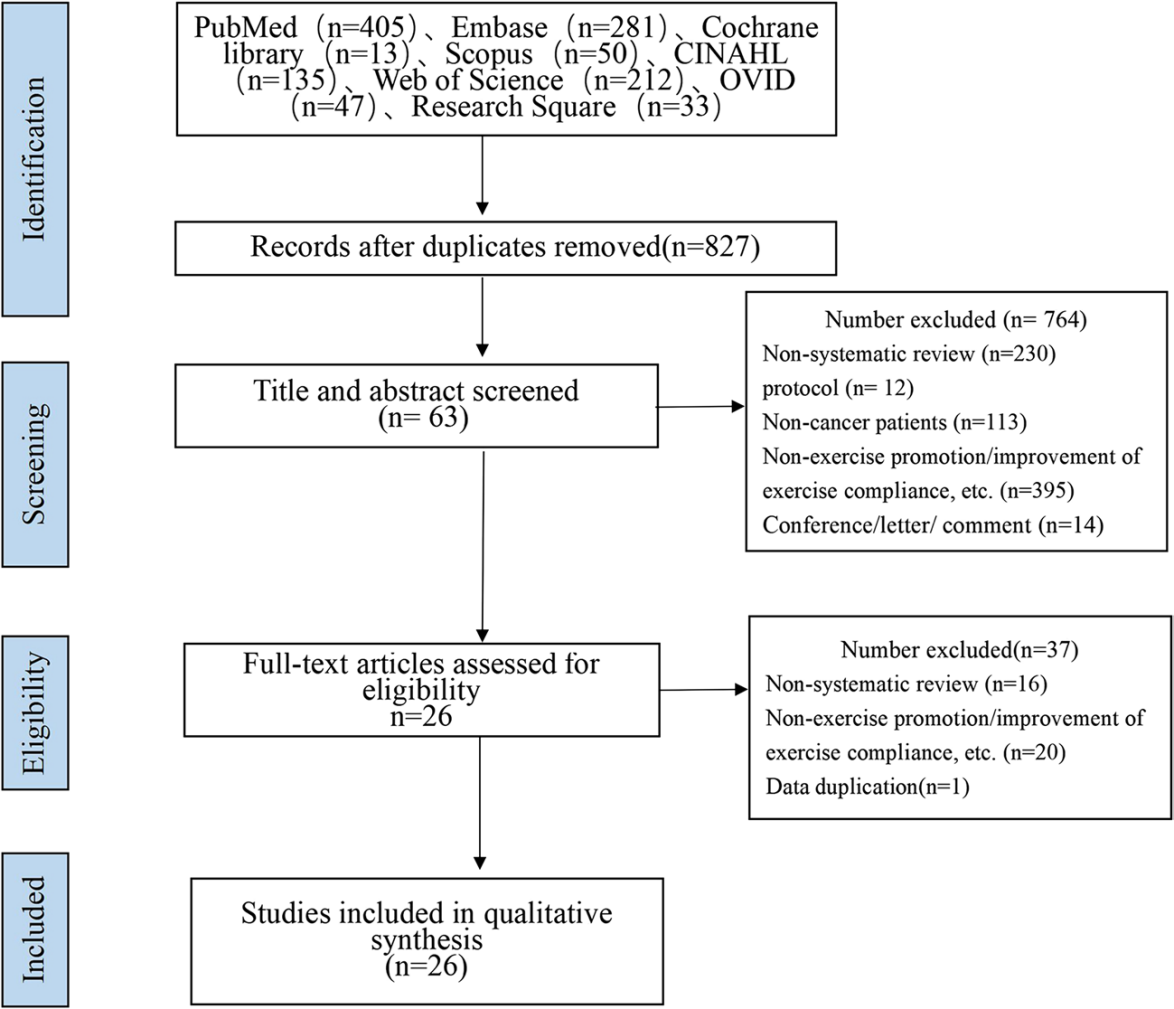
Flow chart of literature search and article selection process

The initial search resulted in 1129 records through database searching; after the removal of duplicates, 820 records remained. Of these, excluded after title, abstract, and full-text screening according to the selection criteria, resulting in 26 articles. (Fig. 1).

### Study characteristics

Overall, the 26 systematic reviews included 67,477 breast cancer patients from 8 countries; included studies were conducted in 8 different countries, mainly in the USA (7/26) and the UK (6/26). Sixteen of these study designs were all in randomized controlled trial. Thirteen studies performed meta-analyses. For the evaluation of the risk of bias, 18 of included studies used the Cochrane risk of the bias assessment tool, 2 used the PEDro scale, 2 used the EPHPP tool, 3 used other tools, and 1 was unclear that not reported for evaluation. The cancer population involved in the study is mainly breast cancer patients. Eight reviews focused on breast cancer survivors only [9, 14-20], sixteen reviews involved survivors with different types of cancer [21-36], and the remaining 2 reviews targeted survivors of colorectal cancer [37, 38] and pediatric cancer. Participants were completed cancer treatment or on remission phase in 10 reviews [9, 14, 15, 17-21, 23, 38], and the remaining reviews involved participants undergoing or completed cancer treatment [16, 22, 24-37]. The intervention measures to promote the movement of cancer patients include theoretical guidance, behavioral change technology, electronic technology intervention, and provision of counseling, print materials, etc. More detail on the basic characteristics of the included literature is shown in Table 1, and PICO information on included studies can be found in Table S1.

**Table 1:** Studies characteristics

Author	Objective	Country	No. of studies /participants included	Study designs	Analysis method	Quality assessment tools
Short et al. 2013 [14]	to examine the efficacy of behavioral interventions for promoting physical activity among post-treatment breast cancer survivors	Australia	10/1299	RCTs	Descriptive	EPHPP tool
Bluethmann et al. 2015 [15]	to describe the characteristics of PA behavior interventions for BCS, including targeted populations, intervention features, and use of behavior theory and to determine effect size estimates for behavior change from these PA interventions	USA	14/2140	RCTs	Meta-analysis	A 10-point scale based on CONSORT checklist
Dennett et al. 2017 [21]	To evaluate the effectiveness of psychoeducational interventions added to exercise rehabilitation programs for cancer survivors	Australia	2/179	RCTs	Meta-analysis	the PEDro scale
Roberts et al. 2017 [22]	to assess their efficacy in promoting PA, reducing sedentary behavior or improving dietary quality; to explore any effects of DBCIs on BMI/weight, other cancer-relevant outcomes and the theoretical underpinning of included studies	UK	15/1335	RCTs (*n* = 8) Pre-post-studies (*n* = 7)	Meta-analysis	Cochrane’s risk of bias tool
Finne et al. 2018 [23]	to investigate how PA can be effectively promoted in cancer survivors	Germany	30/4507	RCTs	Meta-analysis	EPHPP-Tool
Haberlin et al. 2018 [24]	to explore the effects of eHealth in the promotion of PA among cancer survivors	Ireland	10/1994	RCTs (n = 7) Non-controlled trials (*n* = 3)	Descriptive	The Downs and Black tool
Rossi et al. 2018 [25]	To determine whether theory-based PA interventions for overweight and obese female cancer survivors lead to increased PA and improved health	USA	10/1351	RCTs	Descriptive	An adapted version of previously developed criteria by authors
Turner et al. 2018 [26]	To assess the effects of interventions designed to promote exercise behavior in sedentary people living with and beyond cancer	UK	23/1372	RCTs	Meta-analysis	Cochrane’s risk of bias tool
Abdin et al. 2019 [16]	To examine the effect of PA interventions to improve health outcomes and show whether an increase in PA was achieved by interventions	UK	17/2208	RCTs	Descriptive	Cochrane’s risk of bias tool
Grimmett et al. 2019 [27]	To evaluates the effectiveness of interventions in supporting maintenance of physical activity behavior change among adults diagnosed with cancer and explores which intervention components and contextual features are associated with effectiveness	UK	27/5792	RCTs	Meta-analysis	Cochrane’s risk of bias tool
Schaffer et al. 2019 [28]	Examining the feasibility of E-DATs in cancer survivors and effects on activity level, body composition, objective fitness outcomes, HRQoL, self-reported symptoms, and biomarkers	USA	12/1450	RCTs	Descriptive	No assessment reported
Sheeran et al. 2019 [27]	To quantify the effectiveness of interventions in promoting physical activity among cancer survivors, and determine what combination of change techniques and intervention features is associated with greater effectiveness	USA	138/13050	RCTs	Meta-analysis	Cochrane’s risk of bias tool
Brunet et al. 2020 [30]	To synthesize knowledge regarding the effect of health care provider-delivered physical activity interventions on cancer survivors’ physical activity behavior	Canada	17/1893	RCTs	Descriptive	Cochrane’s risk of bias tool
Dorri et al. 2020 [17]	To investigate the effects of PA interventions provided through eHealth on breast cancer patients	Iran	16/2304	RCTs (*n* = 8), randomized trial without control group (*n* = 3), pre-/post-studies (*n* = 5)	Descriptive	Cochrane’s risk of bias tool
Mbous et al. 2020 [37]	To present and critically discuss the impact of PA interventions in terms of their effectiveness, and impact on moderate-to-vigorous physical activity behavior change	USA	10/2364	RCTs	Meta-analysis	Cochrane’s risk of bias tool
Blackwood and Rybicki 2021 [31]	To describe the type of technology used and outcomes of telehealth-delivered PA programs in adult cancer survivors	USA	5/682	RCTs (*n* = 4), single-arm study (*n* = 1)	Descriptive	Cochrane’s risk of bias tool
Blount et al. 2021 [18]	To establish a more current understanding of the findings in this field of inquiry, potential biases, areas for improvement, and future directions of investigation	USA	14/944	RCTs (*n* = 13), controlled trials (*n* = 1)	Descriptive	Cochrane’s risk of bias tool
Cheung et al. 2021 [38]	To collate evidence and evaluate the effects of physical activity interventions on physical activity level among pediatric cancer survivors who had completed active cancer treatment	China	8/673	RCTs	Descriptive	Cochrane’s risk of bias tool
Ester et al. 2021 [32]	To provide a comprehensive, updated overview of evidence on eHealth PA interventions for adults with cancer	Canada	67/6655	RCTs (*n* = 45), nonrandomized single or two-arm trials (*n* = 22)	Descriptive	Cochrane’s risk of bias tool
Ibeggazene et al. 2021 [33]	To assess the most recent evidence of the effects of remote exercise interventions in people living with and beyond cancer	UK	3/186	RCTs	Meta-analysis	Cochrane’s risk of bias tool
Khoo et al. 2021 [34]	To identify, evaluate, and synthesize the scientific literature on mHealth interventions to promote PA or reduce SB in cancer survivors. To identify, evaluate, and synthesize the scientific literature on mHealth interventions to promote PA or reduce SB in cancer survivors	Singapore	31/1977	RCTs (*n* = 16), controlled trials (*n* = 3), pre-and-post trials (*n* = 12)	Descriptive	Cochrane’s risk of bias tool
Liu et al. 2021 [19]	To evaluate the effects of behavior change theory-based physical activity interventions for women with breast cancer and the application of these theories	Australia	19/1877	RCTs, quasi-randomized controlled study	Meta-analysis	Cochrane’s risk of bias tool
Meyer-Schwickerath et al. 2021 [35]	To assess whether cancer survivors maintain or even increase their physical activity behavior after participating in a face-to-face behavior change counseling intervention, to answer the question which behavior change techniques are most effective in this target group and to compare interventions and theoretical foundation	Germany	14/1666	RCTs	Meta-analysis	The PEDro scale
Pudkasam et al. 2021 [20]	To compare adherence to self-directed PA in programs applying a step tracker or MI in female breast cancer survivors and identify whether either of these behavioral change techniques is more effective in promoting adherence to self-directed PA in breast cancer survivors	Australia	16/1668	RCTs	Meta-analysis	Cochrane’s risk of bias tool
Hailey et al. 2022 [9]	To advance our understanding of which behavior change techniques have been used in interventions promoting breast cancer survivors’ PA and to evaluate their potential to increase PA	UK	27/3656	RCTs (*n* = 25), quasi experimental studies (*n* = 1), Quasi RCT (*n* = 1)	Descriptive	Cochrane’s risk of bias tool
Singh et al. 2022 [36]	To evaluate the effect of wearable devices for improving physical activity and health-related outcomes in cancer survivors	Australia	35/4255	RCTs	Meta-analysis	Cochrane’s risk of bias tool

*PA* physical activity, *BCS* breast cancer survivors, *DBCIs* digital behavior change interventions, *BMI* body mass index, *E-DATs* digital activity trackers, *HRQoL* health-related quality of life, *SB* sedentary behavior, *RCTs* randomized controlled trials

## Methodological quality and evidence grade evaluation

The methodological quality evaluation results showed that 1 study was high, 10 were intermediate, 9 were low, and 6 were very low. Among the 7 key items of AMSTAR 2 quality evaluation, item 2 and item 15 have significant defects; the non-critical items with obvious defects are item 3 and item 10 (details in Figure S1). The results of the level of evidence evaluation showed that 16 articles were ranked as 1a, that is, systematic reviews of multiple RCTs; 10 articles were ranked as 1b, that is, systematic reviews of multiple RCTs and other interventional studies.

## Summary of exercise promotion measures (Table 2)

**Table 2:** Main results and conclusions of the included literature

Author	Conclusions on Effectiveness	Main results and conclusions	Evidence level^*^
Systematic review and meta-analysis			
Bluethmann et al. 2015 [15]	Moderate-intensity activities plus various behavioral change strategies had positive effects on behavior changes in physical activity in a meta-analysis of 14 studies (SMD = 0.47 (Cohen’s *d*); 95% CI: 0.23 to 0.67; *p* < 0.001; *I*^2^ = 76.1%)	The mean duration of trials was 17 weeks. All interventions included moderate-intensity activities plus various behavioral change strategies. Most interventions were partially or entirely home-based. Highly structured interventions tended to have larger behavioral change effects, with larger effect from those with telephone counselling or email support. “More” directly supervised/monitored physical activity behavioral interventions may not be better	1a
Dennett et al. 2017 [21]	Psychoeducation and exercise intervention were combined in a meta-analysis of 2 studies reporting no statistical difference (SMD = 0.05; 95% CI = 0.25–0.36; *p* = 0.72; *I*^2^ = 0)	Intervention length was 4 weeks and 12 weeks. There is insufficient evidence to support adding psychoeducation to an exercise rehabilitation program for improving physical activity behavior or improving health outcomes in cancer survivors	1a
Roberts et al. 2017[22]	DBCIs resulted in significant increases in MVPA minutes/ week in a meta-analysis of 7 studies (MD = 41.47; 95% CI = 12.17–70.77; *p* = 0.006; *I*^2^ = 81%)	The length of interventions ranged from 4 weeks to 6 months. DBCIs may improve PA and BMI among cancer survivors. Self-monitoring of behavior, goal setting (behavior), credible source and feedback on behavior were the most frequently described BCTs	1b
Finne et al. 2018 [23]	• BCTs had a significant effect on PA level in a meta-analysis of 30 studies (g = 0.28; 95% CI = 0.18–0.37; *I*^2^ = 54.29%) • Effect sizes did not increase meaningfully with the number of BCTs per intervention (β = 0.005, 95% CI = − 0.007 to 0.017, *P* = 0.408) • Interventions based on the TPB revealed significantly smaller effects (β = − 0.126, 95% CI = − 0.184 to 0.067, *P* ≤ 0.001) • Interventions that included a home-based training component seemed more effective than clinics or gyms in a meta-analysis of 27 studies (β = 0.261, 95% CI = 0.135–0.386, *P* ≤ 0.001) • Home-based and Facility combined showed the largest effect sizes in a meta-analysis of 14 studies. (β = 0.386, 95% CI = 0.281–0.491, *P* ≤ 0.001)	The duration of interventions ranged from one-time recommendation to 12 months. Certain BCTs were associated with an increase of PA in cancer survivors. The BCTs Prompts, reduce prompts, Graded tasks, Non-specific reward, and social reward were significantly related to larger effects, while Information about health consequences and Information about emotional consequences, as well as social comparison were related to smaller effect sizes Interventions based on the Theory of Planned Behavior were associated with smaller effect sizes, and interventions with a home-based setting component were associated with larger effect sizes	1a
Turner et al. 2018 [26]	• Promote exercise behavior: no meta-analysis • Interventions resulted in improvements in aerobic exercise tolerance at 8 to 12 weeks (SMD = 0.54, 95% CI = 0.37–0.70) • At six months, aerobic exercise tolerance was also improved (SMD = 0.56, 95% CI = 0.39–0.72)	Most studies were around 8 to 12 weeks The most frequent of these interventions were setting of graded tasks, program set goal and instruction on how to perform behavior. Setting goals, graded physical activity tasks and providing instructions on how to perform the exercises could help people to do beneficial amounts of exercise. These studies were predominantly exclusively supervised studies or a combination of supervised and home-based studies. Supervision usually consisted of contact with the exercise professional or research team at least twice weekly	1a
Grimmett et al. 2019 [27]	Intervention group with a small estimated effect in a meta-analysis of 19 studies (SMD = 0.25; (95% CI = 0.16–0.35); *I*^2^ = 36%)	The length of the interventions varied from a single contact to 10 months of regular interactions. Existing interventions are effective in achieving modest increases in physical activity at least 3 months post-intervention completion. Reasonably low-intensity interventions may be sufficient in prompting lasting behavior change in motivated, young, well-educated and white populations but that more intensive support is likely to be required for other populations, especially for older people and those with physical limitations	1a
Sheeran et al. 2019 [29]	• Using prompt specific goal setting in the control condition was associated with a smaller intervention effect in a Meta-regression of 22 studies (B = − 0.25, SE = 0.09, *p* = 0.006) • Involved supervised exercise sessions were associated with greater effectiveness in a meta-regression of 54 studies (B = 0.20, SE = 0 .07, *p* = 0.005) • Overweight or engaged in little physical activity had larger effect sizes in a Meta-regression of 84 studies (B = 0.15, SE = 0.07, *p* = 0.03) • Interventions set in community centers and other settings (i.e., not home, clinic, or other community settings) were associated with larger effects in a Meta-regression of 17 and 9 studies (B = 0 .20 and 0.36, SE = 0.10 and 0.17, respectively, *p* = 0.05) • Interventions delivered by mail and interventions involving self-complete or tailored workbooks exhibited smaller effects compared to other modes of delivery in a Meta-regression of 27 and 13 studies (B = − 0.18 and − 0.29, SE = 0.08 and 0.10, ps < 0.03) • Greater contact time was associated with increased effectiveness in a Meta-regression of 63 studies (B = 0.002, SE = 0.001, *p* = 0.008)	Follow-up periods for interventions ranged from immediate to 4.78 years (M = 12.26 weeks). Interventions were conducted at home (n = 91), in hospital/clinic settings (*n* = 41), and/or community centers (n = 17), The most frequently deployed techniques in treatment conditions were: Prompt specific goal setting (*n* = 108), Prompt self-monitoring of behavior (n = 81), Prompt intention formation (*n* = 66), and Prompt barrier identification (*n* = 63) Meta-CART analysis indicated that the major difference in effectiveness was attributable to supervised versus unsupervised programs. And longer supervised programs had an effect of medium-to-large magnitude. For unsupervised programs, establishing outcome expectations, greater contact time, and targeting overweight or sedentary participants each predicted greater program effectiveness, establishing outcome expectations would seem to be a key ingredient for increasing physical activity in unsupervised programs, whereas prompting barrier identification and providing workbooks were associated with smaller effect sizes. Higher BMI was associated with greater intervention effectiveness and interventions that deliberately targeted participants who are overweight or sedentary were especially effective	1a
Ibeggazene et al. 2021 [33]	• Promote exercise behaviors: no meta-analysis • Aerobic exercise tolerance was unchanged in the intervention versus the control at 12 weeks (SMD = 0.70, 95% CI = 0.37–1.03)	All trials delivered their interventions over an initial 12-week period. There is little evidence suggesting that remote exercise interventions promote exercise behaviors or improve physical function in sedentary adults living with and beyond cancer	1a
Mbous et al. 2020 [37]	Theory-based PA interventions among CRC survivors improve physical activity in a meta-analysis of 8 studies (ES = 0.26, 95% CI = 0.13–0.38, *I*^2^ = 16%)	Mean intervention length was 10 months. The transtheoretical model (*n* = 3), social cognitive theory (*n* = 3), and theory of planned behavior (*n* = 2) were the most used theories. “goal setting (behavior)” (*n* = 10), “goal setting (outcome)” (*n* = 10), “action planning” (*n* = 9), and “problem solving” (*n* = 9) were the most commonly used BCTs. Intervention modalities were primarily print material based (*n* = 4) and telephone counseling (*n* = 4). Theory-based PA interventions for colorectal cancer survivors are effective in improving PA uptake among CRC survivors	1a
Liu et al. 2021 [19]	• Behavior change theories had medium improvements in self-reported physical activity in a meta-analysis of 12 studies (SMD = 0.57, 95% CI = 0.33–0.80, *I*^2^ = 67%) • Objectively measured physical activity in a meta-analysis of 7 studies (SMD = 0.52, 95% CI = 0.14–0.89, *I*^2^ = 75%)	Intervention length ranged from 12 weeks to 8 months (mean = 16.5 ± 7.1 weeks). Most trials cited the social cognitive theory (*n* = 10) and transtheoretical model (*n* = 9). Trials rarely applied theories in their entirety, expounded on behavioral mechanisms, or tailored interventions according to behavioral constructs. The most commonly used types of behavioral techniques were goals and planning (*n* = 18), shaping of knowledge (*n* = 18), feedback and monitoring (*n* = 17), and comparisons of outcomes (*n* = 17). Behavior change theories were effective for increasing physical activity in women with breast cancer	1b
Meyer-Schwickerath et al. 2021 [35]	Face-to-face behavior change counseling interventions improved physical activity behavior in a meta-analysis of 13 studies (SMD = 0.22, 95%CI = 0.11–0.33, *I*^2^ = 6%)	Most interventions were six months in duration and ranged from one contact (exercise recommendation) to 12 months. The BCTs “graded tasks,” “self-monitoring of behavior,” “action planning,” and “habit reversal” were more frequently coded in more efficacious interventions. Behavior change counseling interventions are effective in increasing PA behavior in cancer survivors. The behavior change technique “Graded tasks” appears to be a promising approach to make face to-face behavior change counseling interventions more effective	1a
Pudkasam et al. 2021 [20]	• Motivational strategies improved MVPA duration in a meta-analysis of 10 studies (SMD = 0.55, 95% CI = 0.30–0.79; *I*^2^ = 51.4%) • Motivational strategies interventions meeting PA recommendation in a meta-analysis of 7 studies (OR = 2.66, 95% CI = 1.34–5.2; *I*^2^ = 77%)	All trials had a median duration of follow-up of 12 weeks. Pedometer combined with another intervention has a small effect on step count (*p* = 0.03) and a moderate effect on duration of moderate-vigorous physical activity (MVPA) (*p* = < 0.0001) compared to controls. Motivational strategies increase the number of participants who meet a PA goal (*p* = 0.005). Step tracker combining with counselling, print material or MI based on behavioral change theory provided a consistent positive effect on adherence to self-directed PA among breast cancer survivors	1a
Singh et al. 2022 [36]	• Physical activity tracker and pedometer-based interventions had moderate-to-large effects (all *p* < 0.05) on duration of moderate-intensity physical activity in a meta-analysis of 15, 16, 19 studies (SMD = 0.87, 95% CI = 0.43–1.32), MVPA (SMD = 0.61, 95% CI = 0.36–0.86), total physical activity (SMD = 0.62, 95% CI = 0.39–0.84) and daily steps (SMD = 0.54, 95% CI = 0.30–0.78) • Interventions with baseline counselling had larger effects on moderate-intensity physical activity (yes: SMD = 1.13; no: SMD = 0.26; *x*^2^ = 5.69, *p* = 0.02), MVPA (yes: SMD = 0.99; no: SMD = 0.26; x^2^ = 8.48, *p* < 0.01), and daily steps (yes: SMD = 0.72; no: SMD = 0.09; *x*^2^ = 11.43, *p* < 0.01) • Greater increases in MVPA were observed following interventions 12 weeks (SMD = 0.73) in duration compared with interventions < 12 weeks (SMD = 0.19; *x*^2^ = 4.78, *p* = 0.03) in duration • Theory-based interventions (SMD = 0.93) had larger effects on total physical activity than non theory based interventions (SMD = 0.40; *x*^2^ = 6.05, *p* = 0.01)	Intervention durations ranged between 4 weeks and 1 year. Most trials (*n* = 25, 71%) involved pedometer-based physical activity interventions. Seven (20%) involved Fitbit-based interventions, and 3 (9%) involved other wearable physical activity trackers. Compared to usual care, wearable devices had moderate-to-large effects on moderate-intensity physical activity, moderate-to-vigorous-intensity physical activity, total physical activity and daily steps. Compared to usual care, those in the intervention had higher quality of life, aerobic fitness, physical function and reduced fatigue. Wearable physical activity trackers and pedometers are effective tools that increase physical activity and improve health-related outcomes in individuals with cancer	1a
Systematic review			
Short et al. 2013 [14]	Eight out of the ten identified studies reported positive intervention effects on aerobic physical activity behavior	The most frequent duration of the interventions was 12 weeks. The most commonly employed behavior change techniques were self-monitoring and goal-setting, eliciting social support and positive reinforcement. 12-week interventions employing behavior change techniques derived from a variety of theories and delivered in a variety of settings (i.e., one-on-one, group or home) can be effective at changing the aerobic physical activity behavior of breast cancer survivors in the mid- to long terms	1b
Haberlin et al. 2018 [24]	All studies reported improvements in PA, with 8/10 studies reporting statistically significant changes	The length of interventions ranged from 14 days to 12 months. eHealth interventions may increase PA in cancer survivors, with the majority of studies (8/10) reporting improvements in PA and exercise	1b
Rossi et al. 2018 [25]	Of the 8 studies that administered home-based combined with center-based interventions, 5 observed significant improvements in self-reported physical activity, pedometer or accelerometer physical activity counts, or both. Home-based interventions led to small improvements in PA (Cohen’s d range = 0.25–0.31), whereas home-based plus center-based interventions led to moderate to large improvements (Cohen’s d range = 0.45–1.02)	Intervention length ranged from 12 weeks to 1 year Theory-based PA interventions are safe and feasible for overweight and obese female cancer survivors. Home-based plus center-based physical activity interventions may increase physical activity more than home-based programs alone among overweight and obese female cancer survivors	1a
Abdin et al. 2019 [16]	6 of 17 trials demonstrated significant intervention effects were maintained	The length of reporting ranged from a minimum of 12 weeks up to 2 years. Current findings suggest that both group and individual PA interventions for individuals with breast cancer have positive outcomes	1a
Schaffer et al. 2019 [28]	Compared with controls, E-DATs significantly improved patients’ step count in 3 of 5 RCTs, activity level in 6 of 9 RCTs (all *P* ≤ .05)	Duration of E-DATs ranged from 4 to 24 weeks E-DATs are feasible to implement in cancer survivors, with the potential to positively impact numerous clinically meaningful outcomes	1a
Brunet et al. 2020 [30]	• Two studies reported within person increases in physical activity behavior and one reported decrease • Nine studies reported between-group differences in physical activity behavior favoring the intervention group	Mean intervention length was 15 weeks, and the most frequent duration of the interventions was 12 weeks. Seven of these used the social cognitive theory, one used the theory of planned behavior, and one used self-management principles. Two studies used motivational interviewing and narrative therapy. The most commonly used BCTs were “goal setting—behaviors” (*n* = 7), “:self monitoring of behavior” (*n* = 5), and “problem-solving” (*n* = 4) Health care provider-delivered physical activity interventions may help to increase cancer survivors’ physical activity behavior	1a
Dorri et al. 2020 [17]	The majority of studies (10/16) reported positive effects on PA (*p* < 0.05)	The technology used in included studies comprised web based, mobile-based (apps), web-and-mobile-based apps as well as email. The mobile apps used were Lose it!, Smart After Care, GAINFitness, MapMyFitness, and Fitbit The use of eHealth tools is effective in promoting PA in BC patients and can be used as a supportive opportunity for these patients	1b
Blackwood and Rybicki 2021 [31]	Only 2 studies reported significant differences in PA from baseline to post-intervention	Exercise/PA program varying from 8 weeks to 8 months. PA interventions were delivered via telehealth by telephone calls (2 studies), smartphone apps (2 studies), and one used a combination of telephone calls and text messaging. Limited evidence exists describing better outcomes of telehealth-delivered PA programs in cancer survivors than by traditional home exercise program instruction	1b
Blount et al. 2021 [18]	• Eight studies of the positive relationships observed were statistically significant • Six studies which utilized multi-component interventions generally yielded more significant results	Most studies were a 12-week trial. The Fitbit One, Polar A360/M400, activPAL, and Garmin Vivofit smartwatches were most popular as an intervention component. increased moderate-to-vigorous intensity physical activity was observed to be associated with increased perceived cognition and higher cognitive performance. Wearable health technology-based physical activity interventions to be effective for improving physical activity, attitude, and cognitive functions and for reducing sedentary behavior, anxiety, and worry in BCS	1b
Cheung et al. 2021 [38]	All studies(8) investigated interventions for pediatric cancer survivors to increase their physical activity level	The intervention period lasted between 6 weeks and 6 months. eHealth and mHealth interventions showed effectiveness and feasibility to promote physical activity among pediatric cancer survivors	1a
Ester et al. 2021 [32]	Significant post-intervention PA increases were noted in 52% (35/67) of the studies	Across studies with a median of 12 weeks. Social cognitive theory (23/67, 34%) was the most used theory. The mean number of BCTs used across the studies was 13.5 (SD 5.5), with self-monitoring, credible sources, and goal setting being used in > 90% of studies. Weight analyses showed the greatest associations between increased PA levels and PA as a primary outcome, interventions using websites or mobile apps, interventions integrating multiple behavioral theories, and interventions using BCTs of problem solving and action planning A range of eHealth PA interventions may increase PA levels among adults affected by cancer, and specific components (e.g., websites, use of theory, and action planning) may be linked to greater effectiveness	1b
Khoo et al. 2021 [34]	• Seven (7/8) reported significant effects of mHealth interventions with personal contact on MVPA • Six non-controlled trials reported on MVPA, of which two reported a significant effect favoring the mHealth intervention • Four studies (4/31) reported a significant decrease in SB following an mHealth intervention	Researchers that assessed an mHealth intervention and its effects on MVPA in controlled studies were often 12 weeks long. Activity trackers being the most commonly used mHealth technology. There is strong evidence for mHealth interventions, including personal contact components, in increasing moderate-to-vigorous intensity PA among cancer survivors. However, there is inconclusive evidence to support mHealth interventions in increasing total activity and step counts	1b
Hailey et al. 2022[9]	Potential of the intervention to increase PA behavior; “very promising” (*n* = 11), “quite promising” (*n* = 13)	Interventions lasted from 8 to 104 weeks. Demonstration on how to perform the behavior was the most commonly used BCT (*n* = 23). Adding objects to the environment (pedometer or accelerometer) was the BCT with the highest potential to increase PA. This was followed by goal setting and self-monitoring of behavior. “Instructions on how to perform the behavior,” “goal setting,” “self-monitoring,” and adding a pedometer or accelerometer into the environment in combination appear to have a beneficial effect on PA. BCTs have the potential to increase PA for breast cancer survivors and inform intervention development	1b

*SMD* standardized mean difference, *MD* mean difference, *DBCIs* digital behavior change interventions, *MVPA* moderate to vigorous physical activity, *PA* physical activity, *BMI* body mass index, *BCTs* behavior change techniques, *CRC* colorectal cancer, *TPB* theory of planned behavior, *E-DATs* digital activity trackers

^*^1a systematic reviews of multiple RCTs, 1b systematic reviews of multiple RCTs and other interventional studies

### Intervention settings

Intervention settings in our reviews included studies that included group, one on one, home, clinic or research-setting, and center-based. Most reviews included studies that were mainly delivered in home settings [9, 15, 19, 20, 23-25, 27-29, 38]. Some reviews that delivered in a variety of settings included a combination of supervised and home-based exercise, supervised combined with unsupervised [14, 16, 19, 26, 30]. In reviews that focused on a single intervention setting, all were conducted in a home setting [18, 33]. Home-based exercise usually has behavioral support, including information feedback, ongoing interaction, or counseling with the research team. Five reviews did not report the setting [17, 32, 34, 36, 37]. Interventions that included a home-based training component seemed more effective than clinics or gyms in a meta-analysis of 27 studies (β = 0.261; 95% CI = 0.135, 0.386; *P* ≤ 0.001) [23]. And the review by Rossi et al. [25] indicated that home-based combined center-based interventions may increase physical activity more than home-based programs alone among overweight and obese female cancer survivors.

### Intervention length

The intervention length varied from 2 weeks to 4.78 years. The most frequent and mean duration of the interventions was 12 weeks [14, 18, 20, 29, 30, 32, 34]. Four reviews included the length of reporting ranging from a minimum of 12 weeks [16, 19, 25, 33], four reviews included the length of interventions ranging to 12 months [23, 24, 35, 36]. The studies included a one-time recommendation, or follow-up during intervention and post-intervention, as Grimmett et al. [27] included a physical activity behavior follow-up assessment ranging from 3 months post-intervention to 5 years post-intervention. The review by Singh et al. [36] indicated that interventions lasting 12 weeks (SMD = 0.73) were greater at increasing MVPA compared with interventions < 12 weeks (SMD = 0.19; *x*^2^ = 4.78, *p* = 0.03). And interventions are effective for a moderate increase in physical activity at least 3 months after the intervention (interventions detail in Table S1)[27].

### Physical activity measures

Most studies used self-reported measures to assess the patients’ physical activity behavior. Nineteen reviews (73.07%) [14, 15, 17, 19, 21-25, 27, 28, 30-35, 37, 38] involved subjective PA measures; the subjective PA measurement tools used were mainly the Godin Leisure Time Exercise Questionnaire (GLTEQ), International Physical Activity Questionnaire (IPAQ), 7-day physical activity recall (7-DPARQ), Godin Leisure score index, Scottish physical activity questionnaire (Scot-PASQ), the Short Questionnaire to Assess Health Enhancing Physical Activity (SQUASH), 7-day PA and Community Health Activities Model Program for Seniors (CHAMPS), etc. And some studies used a self-log method. Fourteen reviews (53.84%) [14, 17-19, 23-27, 32-35, 38] involved objective PA measures (i.e., accelerometers, Fitbit, and pedometers). The objective measurement tools are small and light; these can be worn on the waist or wrist during waking hours. Seven reviews did not clearly report the measures [9, 16, 20, 23, 29, 34, 36].

### Types of interventions and their effectiveness on physical activity (Table S1)

#### Electronic interventions

Investigating the effect of electronic interventions on cancer survivors were 34.61% (*n* = 9) of the total included studies [17, 18, 22, 24, 28, 31, 32, 34, 36, 38]. Of these, seven studies aimed at the effect of electronic health (eHealth) on exercise levels in cancer patients [17, 22, 24, 31, 32, 34, 38]. eHealth uses technologies including telephones, websites, email, and mobile health (mHealth) technologies. The majority of studies (6/7) [17, 22, 24, 32, 34, 38] reported that eHealth interventions were effective and feasible in promoting physical activity in cancer survivors. eHealth resulted in a significant increase in MVPA minutes/week (MD = 41; 95% CI = 12, 71; *p* = 0.006) in a meta-analysis that included 5 RCTs and 2 pre-post studies [22]. Compared with the control interventions, eHealth technology provides communication (including real-time, automated reminders) and feedback.

Three studies were included which aim to examine the feasibility of digital activity trackers in cancer survivors and their effects on activity levels. And activity trackers were the most commonly used mHealth technology [34], and interventions based on physical activity trackers and pedometers usually include BCTs or theory-based interventions [22, 36]. In the included studies, wearable physical activity trackers mainly include Fitbit One, Polar A360/M400, GT3X + ActiGraph, ActivPAL, and Garmin Vivofit smartwatches. Four reviews [18, 28, 31, 34] found that wearable health technology–based physical activity interventions are effective in improving physical activity, and health-related outcomes in individuals with cancer. The review by Khoo et al. [34] indicated there is strong evidence that mHealth includes an individual exposure component to increase moderate intensity PA in cancer survivors. And physical activity tracker and pedometer-based interventions had moderate-to-large effects (all *p* < 0.05) on the duration of moderate-intensity physical activity (SMD = 0.87; 95% CI = 0.43, 1.32), MVPA (SMD = 0.61;95% CI = 0.36,0.86), total physical activity (SMD = 0.62; 95% CI = 0.39, 0.84), and daily steps (SMD = 0.54; 95% CI = 0.30, 0.78) in a meta-analysis of 15, 16, and 19 studies, respectively [36]. Subgroup analysis by Singh et al. [36] showed that baseline counseling (compared to no baseline counseling taken) and theory-based were effective in increasing PA levels, respectively.

#### Behavior change techniques and theory-based interventions

Among the included studies, five studies were limited to behavioral change techniques (BCTs) and theory-based interventions [9, 19, 20, 23, 25], and five studies related to integrated interventions [14, 26, 30, 35, 37]. BCTs in the included studies were seven [9, 14, 23, 26, 30, 35, 37]. BCTs had a significant effect on PA levels in a meta-analysis of 30 studies (*g* = 0.28; 95% CI = 0.18, 0.37; *I*^2^ = 54.29%)[23]. Motivational strategies improved MVPA duration in a meta-analysis of 10 studies (SMD = 0.55; 95% CI = 0.30, 0.79; *I*^2^ = 51.4%) [20]. The most commonly used BCTs were goal setting (behavior), goal setting (outcome), action planning, habit reversal, instruction on how to perform the behavior, self-monitoring, eliciting social support, positive reinforcement, and problem-solving. And effect sizes did not increase meaningfully with the number of BCTs per intervention (β = 0.005; 95% CI =  − 0.007, 0.017; *P* = 0.408) [23]. The BCTs prompts, reduce prompts, graded tasks, non-specific rewards, and social rewards were significantly associated with larger effects, while information about emotional consequences and social comparison was associated with smaller effects size [23]. Face-to-face behavior change counseling interventions improved physical activity behavior in a meta-analysis of 13 studies (SMD = 0.22; 95% CI = 0.11,0.33; *p* < 0.001; *I*^2^ = 6%) [35]. Supervision is most important for adherence [26], and involved supervised exercise sessions were associated with greater effectiveness in a meta-regression of 54 studies (B = 0.20, SE = 0.07, *p* = 0.005) [29].

Four reviews involved theory-based behavioral interventions [19, 25, 30, 37]. Most studies cited the social cognitive theory, the transtheoretical model, and the theory of planned behavior. Most reviews suggest that theory-based PA interventions can increase PA in cancer survivors. Theory-based PA interventions among colorectal cancer survivors improve physical activity in a meta-analysis of 8 studies (ES = 0.26; 95% CI = 0.13, 0.38; *I*^2^ = 16%) [37]. Behavior change theories had medium improvements in self-reported physical activity in a meta-analysis of 12 studies (SMD = 0.57; 95% CI = 0.33, 0.80; *I*^2^ = 67%) [19]. And there were smaller effect sizes for TPB-based interventions in a meta-analysis of 7 studies [23]. The included review based on 16 studies [20] showed that the combination of step trackers with counseling, printed materials, or motivational strategies based on behavioral change theory provided a consistently positive effect on adherence to self-directed PA among breast cancer survivors.

#### Psychoeducation

Dennett et al. [21] evaluated the effectiveness of psychoeducational interventions in physical activity behavior change among cancer survivors. This review of 2 randomized controlled trials demonstrated that adding psychoeducation to exercise rehabilitation programs had no additional benefit in improving physical activity behaviors or improving health outcomes in cancer survivors.

#### Other interventions

Five studies were included to assess the effectiveness of interventions in promoting physical activity [15, 16, 27, 29, 38]; interventions in included studies were workshops, group exercise, walking, behavioral counseling, home-based/group-supervised exercise classes, counseling, and group discussions, printed materials and pedometer, etc. Most studies use a combination of interventions for exercise promotion intervention. Reviews indicate that both group and individual PA interventions for individuals have had positive outcomes. Too frequent direct supervision may not be better for physical activity levels. And greater contact time was associated with increased effectiveness in a meta-regression of 63 studies (B = 0.002, SE = 0.001, *p* = 0.008)[29]. Reasonably low-intensity interventions may be sufficient to induce lasting behavioral change in positive, young, well-educated, and white populations, but other populations may require more intensive support, especially older and physically constrained populations. Overweight or sedentary participants had larger effect sizes on PA levels in a meta-regression of 84 studies (B = 0.15, SE = 0.07, *p* = 0.03).

### Adverse events

Seven of the included reviews [21, 24-26, 33, 36, 38] reported the adverse reactions of patients during exercise intervention (26.9%, *n* = 26), and 19 reviews [9, 14-20, 22, 23, 27-32, 34, 35, 37] did not report and mention on adverse events at all. The review of Cheung et al. [38] showed that no major adverse events occurred in the 8 studies involving pediatric cancer survivors. And the study by Haberlin et al. [24] including 10 studies involving eHealth used interventions showed that no adverse effects have been reported. Adverse events included minor joint injuries/soreness [25, 36], recurrence of previous tendinitis [33], plantar fasciitis [26, 36], and falls [36]. In the exercise intervention of overweight and obese women, there is a serious adverse event, a pelvic stress fracture, was reported [25].

## Discussion

Exercise is one of the vital rehabilitation strategies for cancer patients. This umbrella review summarized evidence from systematic reviews and meta-analyses on interventions that improve exercise levels and maintain or improve exercise compliance in cancer patients. The main findings of our umbrella review were that (1) the most frequent and mean duration of the exercise interventions was 12 weeks, and 3 months post-intervention completion can be assessed and guided; (2) electronic, wearable health technology-based, BCTs, and theory-based interventions were effective and feasible in promoting physical activity in cancer survivors; promoting exercise requires a combination of interventions; (3) digital activity trackers were the BCTs with the highest potential to increase PA; (4) the BCTs prompts, reduce prompts, graded tasks, non-specific reward, and social reward were significantly associated with larger effects, while information about emotional consequences and social comparison were associated with smaller effects size; and (5) higher BMI was associated with higher intervention effects, and interventions targeting overweight or sedentary participants were particularly effective.

In this umbrella review, most of the interventions reported statistically significant results for PA outcome in cancer patients. Studies [17, 22, 24, 32, 34, 38] have shown that electronic interventions are expected to improve the level of exercise in cancer patients. Particularly, physical activity tracker and pedometer-based interventions had moderate-to-large effects on duration of moderate-intensity physical activity[36]. eHealth is used as an auxiliary tool for exercise intervention. Most studies [22, 34, 36] on eHealth usually include counseling, educational sessions, personal contact, and BCTs. And baseline counseling is necessary for cancer patients during exercise intervention [35, 36]. There is strong evidence for BCTs in increasing moderate-to-vigorous intensity PA among cancer patients. It is worth noting that not all technologies seem to be applicable, and the effect of intervention may not increase with the increase of the number of BCTs per intervention. BCTs combining with activity trackers is the highest potential to increase PA [23]. Most of the studies show the importance of interaction and behavioral intervention on the exercise behavior of cancer patients, and there are few studies considering the maintenance of exercise behavior after intervention [16, 18, 24, 27, 32]. At the same time, the importance of personal contact components was also found in this umbrella review, but the frequency of assessment or feedback in exercise intervention needs further study.

Although exercise has been advocated, cancer survivors face a series of factors that may impede or facilitate their participation in physical activity. The key barriers to exercise were treatment-related side effects, lack of time, and fatigue [39]. And the critical facilitators of exercise include improved physical health, improved mental well-being, gaining control, and the social benefits of exercise [39]. And fatigue is one of the most commonly reported side effects of cancer treatment [40, 41]. About 70% of cancer patients receiving chemotherapy or radiotherapy have cancer-related fatigue [41]. Many studies have shown that exercise can effectively alleviate the fatigue of patients while improving other adverse reactions, as well as improving physical function and quality of life [42, 43]. However, fatigue is also one of the main obstacle factors for patients’ exercise. Therefore, it is particularly important to improve the exercise level of patients through intervention measures. The study by Machado et al. [44] showed that exercise training is an effective intervention to reduce CRF, especially in patients receiving chemotherapy. Similar to previous studies [45-47], by guiding patients in time management; providing effective, time-saving, and personalized exercise programs; and supporting relevant electronic equipment and network platforms, patients can be promoted to exercise. However, most of the participants in the included systematic review were in the post treatment, and more evidence is needed for measures related to PA level improvement in cancer patients undergoing treatment.

To our best knowledge, this umbrella review is the first to summarize the interventions for promoting physical activity. In similar studies on different populations, most of the physical activity promotion studies focus on children; few studies focus on adolescents or the elderly [48, 49]. In our study, most interventions tend to be a combination of approaches, including electronic, wearable health technology-based, BCTs, theory-based, and combined interventions. Disease treatment seeks targeted intervention, and if exercise intervention can be targeted, it can also maximize the patient’s exercise level and exercise compliance. The study by Rossi et al. [25] found that among overweight and obese female cancer survivors, home-based integration of center-based physical activity interventions may improve patient activity levels more than home-based programs alone. Interventions are more effective in overweight or sedentary patients than in overweight and obese women. Supervision of sedentary patients is the most important measure to improve compliance [26]. However, in terms of interventions for different symptoms, more research is needed to explore the better effectiveness of different interventions.

## Limitation

The quality of the comprehensive review depends on the quality of the included reviews, which in turn depends on the quality of the primary study. Therefore, we need to consider the limitations of the study. First, moderate to high-grade studies were limited as assessed by the AMSTAR 2, mainly related to the study’s protocol design and reporting, and investigation and discussion of publication bias, a common mistake in most systematic reviews. Second, although the incidence of adverse events was low in the reported studies, the included reviews lacked monitoring of the adverse effects of exercise interventions. Finally, the details of the intervention still need further research, including the effect of duration and frequency on patients’ physical activity levels.

## Conclusion

Oriented to positive health and modifiable factors, our study provides an overview of strategies used in the field of exercise promotion, clarifying the intervention setting, duration of intervention, and intervention strategies. It provides an evidence-based basis and reference for clinical practitioners who need rapid access to evidence related to the promotion of exercise in cancer patients. There is abundant evidence that eHealth, wearable health technology-based (combine with theory, BCTs, counseling, etc.), BCTs, and theory-based interventions were effective and feasible in promoting physical activity in cancer survivors. Faced with different groups of patients, such as overweight, sedentary behavior, the elderly, and limited physical activity, appropriate interventions should be adopted according to the characteristics of the population.

## Supplementary Information

Below is the link to the electronic supplementary material.

Supplementary file1 (PDF 90 KB)

Supplementary file2 (DOCX 162 KB)

## Data Availability

We have full control of all systematic review data and can allow the journal to review, if requested.

## References

[1] Sung H, Ferlay J, Siegel RL (2021). Global cancer statistics 2020: GLOBOCAN estimates of incidence and mortality worldwide for 36 cancers in 185 countries. CA Cancer J Clin.

[2] Bray F, Ferlay J, Soerjomataram I (2018). Global cancer statistics 2018: GLOBOCAN estimates of incidence and mortality worldwide for 36 cancers in 185 countries. CA Cancer J Clin.

[3] Posadzki P, Pieper D, Bajpai R (2020). Exercise/physical activity and health outcomes: an overview of Cochrane systematic reviews. BMC Public Health.

[4] Momma H, Kawakami R, Honda T (2022). Muscle-strengthening activities are associated with lower risk and mortality in major non-communicable diseases: a systematic review and meta-analysis of cohort studies. Br J Sports Med.

[5] Segal R, Zwaal C, Green E (2017). Exercise for people with cancer: a clinical practice guideline. Curr Oncol.

[6] Campbell KL, Winters-Stone KM, Wiskemann J (2019). Exercise Guidelines for Cancer Survivors: Consensus Statement from International Multidisciplinary Roundtable. Med Sci Sports Exerc.

[7] Singh B, Spence RR, Steele ML (2018). A Systematic Review and Meta-Analysis of the Safety, Feasibility, and Effect of Exercise in Women With Stage II+ Breast Cancer. Arch Phys Med Rehabil.

[8] Arem H, Sorkin M, Cartmel B (2016). Exercise adherence in a randomized trial of exercise on aromatase inhibitor arthralgias in breast cancer survivors: the Hormones and Physical Exercise (HOPE) study. J Cancer Surviv Res Pract.

[9] Hailey V, Rojas-Garcia A, Kassianos AP (2022). A systematic review of behaviour change techniques used in interventions to increase physical activity among breast cancer survivors. Breast Cancer (Tokyo, Japan).

[10] Lopez P, Galvão DA, Taaffe DR (2021). Resistance training in breast cancer patients undergoing primary treatment: a systematic review and meta-regression of exercise dosage. Breast Cancer (Tokyo, Japan).

[11] Honjo S, Mizunuma H, Soda M (1989). Effect of estrogen replacement therapy on the serum osteocalcin level in the postmenopausal and castrated women. Nihon Sanka Fujinka Gakkai Zasshi.

[12] Shea BJ, Reeves BC, Wells G et al (2017) AMSTAR 2: a critical appraisal tool for systematic reviews that include randomised or non-randomised studies of healthcare interventions, or both. BMJ (Clinical research ed) 358(j4008.10.1136/bmj.j4008 10.1136/bmj.j4008 PMC5833365 28935701

[13] JBI Levels of Evidence and Grades of Recommendation Working Party (2014) JBI LEVELS OF EVIDENCE. https://jbi.global

[14] Short CE, James EL, Stacey F (2013). A qualitative synthesis of trials promoting physical activity behaviour change among post-treatment breast cancer survivors. J Cancer Surviv.

[15] Bluethmann SM, Vernon SW, Gabriel KP (2015). Taking the next step: a systematic review and meta-analysis of physical activity and behavior change interventions in recent post-treatment breast cancer survivors. Breast Cancer Res Treat.

[16] Abdin S, Lavallée JF, Faulkner J (2019). A systematic review of the effectiveness of physical activity interventions in adults with breast cancer by physical activity type and mode of participation. Psychooncology.

[17] Dorri S, Asadi F, Olfatbakhsh A (2020). A Systematic Review of Electronic Health (eHealth) interventions to improve physical activity in patients with breast cancer. Breast Cancer (Tokyo, Japan).

[18] Blount DS, McDonough DJ, Gao Z (2021). Effect of wearable technology-based physical activity interventions on breast cancer survivors’ physiological, cognitive, and emotional outcomes: A systematic review. J Clin Med.

[19] Liu MG, Davis GM, Kilbreath SL (2021). Physical activity interventions using behaviour change theories for women with breast cancer: a systematic review and meta-analysis. J Cancer surviv Res Pract Online Publ.

[20] Pudkasam S, Feehan J, Talevski J (2021). Motivational strategies to improve adherence to physical activity in breast cancer survivors: A systematic review and meta-analysis. Maturitas.

[21] Dennett AM, Shields N, Peiris CL (2017). Does psychoeducation added to oncology rehabilitation improve physical activity and other health outcomes? A systematic review. Rehabil Oncol.

[22] Roberts A, Fisher A, Smith L (2017). Digital health behaviour change interventions targeting physical activity and diet in cancer survivors: a systematic review and meta-analysis. J Cancer Surviv.

[23] Finne E, Glausch M, Exner A-K (2018). Behavior change techniques for increasing physical activity in cancer survivors: a systematic review and meta-analysis of randomized controlled trials. Cancer Manag Res.

[24] Haberlin C, O'Dwyer T, Mockler D (2018). The use of eHealth to promote physical activity in cancer survivors: a systematic review. Support Care Cancer.

[25] Rossi A, Friel C, Carter L (2018). Effects of Theory-Based Behavioral Interventions on Physical Activity Among Overweight and Obese Female Cancer Survivors: A Systematic Review of Randomized Controlled Trials. Integr Cancer Ther.

[26] Turner RR, Steed L, Quirk H (2018). Interventions for promoting habitual exercise in people living with and beyond cancer. Cochrane Database Syst Rev.

[27] Grimmett C, Corbett T, Brunet J (2019). Systematic review and meta-analysis of maintenance of physical activity behaviour change in cancer survivors. Int J Behav Nutr Phys Act.

[28] Schaffer K, Panneerselvam N, PohLoh K (2019). Systematic review of randomized controlled trials of exercise interventions using digital activity trackers in patients with cancer. JNCCN J Natl Compr Cancer Netw.

[29] Sheeran P, Abraham C, Jones K (2019). Promoting physical activity among cancer survivors: Meta-analysis and meta-CART analysis of randomized controlled trials. Health Psychol.

[30] Brunet J, Wurz A, Nader PA (2020). A systematic review summarizing the effect of health care provider-delivered physical activity interventions on physical activity behaviour in cancer survivors. Patient Educ Couns.

[31] Blackwood J, Rybicki K (2021). Outcomes of Telehealth-Delivered Physical Activity Programs in Adult Cancer Survivors: A Systematic Review. Rehabil Oncol.

[32] Ester M, Eisele M, Wurz A (2021). Current Evidence and Directions for Future Research in eHealth Physical Activity Interventions for Adults Affected by Cancer: Systematic Review. JMIR Cancer.

[33] Ibeggazene S, Turner R, Rosario D (2021). Remote interventions to improve exercise behaviour in sedentary people living with and beyond cancer: a systematic review and meta-analysis. BMC Cancer.

[34] Khoo S, Mohbin N, Ansari P (2021). Mhealth interventions to address physical activity and sedentary behavior in cancer survivors: A systematic review. Int J Environ Res Public Health.

[35] Meyer-Schwickerath C, Morawietz C, Baumann FT et al (2021) Efficacy of face-to-face behavior change counseling interventions on physical activity behavior in cancer survivors–a systematic review and meta-analysis. Disability and rehabilitation 1–16. Advance online publication. 10.1080/09638288.2021.1938247 10.1080/09638288.2021.1938247 34261403

[36] Singh B, Zopf EM, Howden EJ (2022). Effect and feasibility of wearable physical activity trackers and pedometers for increasing physical activity and improving health outcomes in cancer survivors: A systematic review and meta-analysis. J Sport Health Sci.

[37] Mbous YP, Patel J, Kelly KM (2020). A systematic review and meta-analysis of physical activity interventions among colorectal cancer survivors. Transl Behav Med.

[38] Cheung AT, Li WHC, Ho LLK (2021). Physical activity for pediatric cancer survivors: a systematic review of randomized controlled trials. J Cancer Surviv Res Pract.

[39] Clifford BK, Mizrahi D, Sandler CX (2018). Barriers and facilitators of exercise experienced by cancer survivors: a mixed methods systematic review. Support Care Cancer.

[40] Bower JE (2014). Cancer-related fatigue–mechanisms, risk factors, and treatments. Nat Rev Clin Oncol.

[41] Bower JE, Ganz PA, Desmond KA (2000). Fatigue in breast cancer survivors: occurrence, correlates, and impact on quality of life. J Clin Oncol.

[42] Gebruers N, Camberlin M, Theunissen F (2019). The effect of training interventions on physical performance, quality of life, and fatigue in patients receiving breast cancer treatment: a systematic review. Support Care Cancer.

[43] Ehlers DK, DuBois K, Salerno EA (2020). The effects of exercise on cancer-related fatigue in breast cancer patients during primary treatment: a meta-analysis and systematic review. Expert Rev Anticancer Ther.

[44] Machado P, Morgado M, Raposo J (2022). Effectiveness of exercise training on cancer-related fatigue in colorectal cancer survivors: a systematic review and meta-analysis of randomized controlled trials. Support Care Cancer.

[45] Fox L, Wiseman T, Cahill D (2019). Barriers and facilitators to physical activity in men with prostate cancer: A qualitative and quantitative systematic review. Psychooncology.

[46] Michael M, Goble J, Hawk M (2021). Reported Barriers Impeding Adherence to a Physical Exercise Program in Patients With Breast Cancer: A Systematic Review. Rehabil Oncol.

[47] Lavallee JF, Abdin S, Faulkner J (2019). Barriers and facilitators to participating in physical activity for adults with breast cancer receiving adjuvant treatment: A qualitative metasynthesis. Psychooncology.

[48] Craike M, Wiesner G, Hilland TA (2018). Interventions to improve physical activity among socioeconomically disadvantaged groups: an umbrella review. Int J Behav Nutr Phys Act.

[49] Barbosa Filho VC, Minatto G, Mota J (2016). Promoting physical activity for children and adolescents in low- and middle-income countries: An umbrella systematic review: A review on promoting physical activity in LMIC. Prev Med.

