# Supplementary material for: Promoting physical activity among cancer survivors: an umbrella review of systematic reviews
Source: Support Care Cancer. 2023 Apr 25;31(5):301. doi: 10.1007/s00520-023-07760-0 (PMC10129958; doi:10.1007/s00520-023-07760-0)
Supplement: Supplementary file 1 — Supplementary file1 (PDF 90 KB) [file 520_2023_7760_MOESM1_ESM.pdf]

Taking Pubmed as an example, the search method combining subject headings and free words is adopted. The search words are adjusted according to the characteristics of the search platform.

Table1 Pubmed

#1	("Neoplasms"[MeSH Terms] OR "Cancer Survivors"[MeSH Terms] OR "cancer survivor"[Title] OR "cancer patients"[Title] OR "cancer"[Title] OR "tumour"[Title]))	3,899,007
#2	("physical activity"[Title/Abstract] OR "exercise"[Title/Abstract] OR "aerobic"[Title/Abstract] OR "strength training"[Title/Abstract] OR "resistance training"[Title/Abstract] OR "walking"[Title/Abstract] OR "Swimming"[Title/Abstract] OR "Yoga"[Title/Abstract] OR "Tai Chi"[Title/Abstract] OR "Qigong"[Title/Abstract])	576,428
#3	(("Behaviour change"[Title/Abstract] OR "Behavior change"[Title/Abstract] OR "Behavi*"[Title/Abstract] OR "maint*"[Title/Abstract] OR "adherence"[Title/Abstract] OR "compliance"[Title/Abstract] OR "chang*"[Title/Abstract] OR "Motivational strategies"[Title/Abstract] OR "eHealth"[Title/Abstract] OR "mHealth"[Title/Abstract] OR "behaviour change techniques"[Title/Abstract] OR "behavior change techniques"[Title/Abstract])	5,631,473
#3	#1 AND #2 AND #3 AND Filters: Meta-Analysis, Systematic Review Sort by: Publication Date	405
