# Supplementary material for: Promoting physical activity among cancer survivors: an umbrella review of systematic reviews
Source: Support Care Cancer. 2023 Apr 25;31(5):301. doi: 10.1007/s00520-023-07760-0 (PMC10129958; doi:10.1007/s00520-023-07760-0)
Supplement: Supplementary file 2 — Supplementary file2 (DOCX 162 KB) [file 520_2023_7760_MOESM2_ESM.docx]

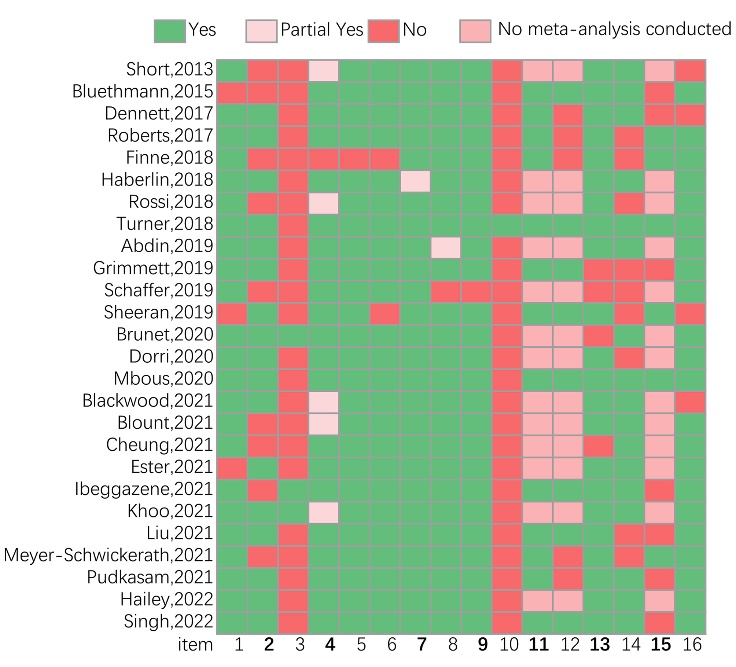


**Figure S1.** Risk of bias assessment with AMSTAR 2. The bold items are key items.

Items 1. Did the research questions and inclusion criteria for the review include the components of PICO? Items 2. Did the report of the review contain an explicit statement that the review methods were established prior to the conduct of the review and did the report justify any significant deviations from the protocol? Items 3. Did the review authors explain their selection of the study designs for inclusion in the review? Items 4. Did the review authors use a comprehensive literature search strategy? Items 5. Did the review authors perform study selection in duplicate? Items 6. Did the review authors perform data extraction in duplicate? Items 7. Did the review authors provide a list of excluded studies and justify the exclusions? Items 8. Did the review authors describe the included studies in adequate detail? Items 9. Did the review authors use a satisfactory technique for assessing the risk of bias (RoB) in individual studies that were included in the review? Items 10. Did the review authors report on the sources of funding for the studies included in the review? Items 11. If meta-analysis was performed did the review authors use appropriate methods for statistical combination of results? Items 12. If meta-analysis was performed, did the review authors assess the potential impact of RoB in individual studies on the results of the meta-analysis or other evidence synthesis? Items 13. Did the review authors account for RoB in individual studies when interpreting/ discussing the results of the review? Items 14. Did the review authors provide a satisfactory explanation for, and discussion of, any heterogeneity observed in the results of the review? Items 15. If they performed quantitative synthesis did the review authors carry out an adequate investigation of publication bias (small study bias) and discuss its likely impact on the results of the review? Items 16. Did the review authors report any potential sources of conflict of interest, including any funding they received for conducting the review?

**Table S1.** Description of PICO components

| **Author** | **Population** | | **Intervention** | **Comparison** | **PA** **outcome measure** |
| --- | --- | --- | --- | --- | --- |
|  | Cancer type | Treatment stage |  |  |  |
| Short,2013 | BCS | post treatment (not including hormone therapy) | Theory**,** BCTs | no intervention, standard/usual care condition, contact control | Self-report in all  pedometer, n=1; accelerometer, n=3 |
| Bluethmann,2015 | BCS | approximately 5 years or less from completion of active cancer treatment | Workshops, group exercise, walking, Behavioral counseling | Na | 7-DPARQ, n=7; GLTEQ, n=3; IPAQ, n=1; Kaiser PA Survey, n=1; PAQ, n=1; CHAMPS, n=1; Self-administered survey, n=1 |
| Dennett,2017 | mixed(n=1), Kidney cancer(n=1) | post treatment | Psychoeducation, supervised exercise program, CBT, counseling | Supervised exercise interventions without a psychoeducation component | PASE, n=1; Modified Leisure Score Index Disease-Specific, n=1 |
| Roberts,2017 | in adults cancer (≥18 years) who had a cancer diagnosis of any type | Undergoing and post treatment | DBCIs, BCTs | Na | GLTEQ, n=5; IPAQ, n=2; 7-day PA, n=1; SQUASH, n=1; percentage of meeting PA guidelines, n=3; resistance training score, n=1; number of minutes walked, n=2 |
| Finne,2018 | breast cancer (n=13), mixed types of cancer (n=13), Prostate(n=4), Uterine(n=1) | post treatment | Theory**,** BCTs; home-based, Facility-based, Combined | Usual care or wait-list control group | Unclear (Objective, n=8; Self−reported, n=30)  steps/day, MVPA min/day, counts (ct/min/day) kcal'total, kcal/week, Leisure Score Index, MET−h/wee |
| Haberlin,2018 | breast(n=5), mixed(n=5) | Undergoing and post treatment | web app(n=5), web and mobile app(n=2), mobile app(n=1), website only(n=1), e-mail based(n=1) | did not receive ehealth intervention, on wait list for intervention | Fitbit, n=1; 7-DPARQ, n=8; GLTEQ, n=2; IPAQ-SF, n=1; Self-reported exercise, SQUASH, n=1; Modifiable Physical Activity Questionnaire, n=1; self-log, n=3 |
| Rossi,2018 | overweight and obese female cancer survivors (breast=7, Breast and colorectal=1, Endometrium =2) | Undergoing and post treatment | Theoretical models, Behavioral Interventions; Home based, Center-based | Usual care | GLTEQ, n=6; 7-DPAR, n=2; accelerometry, n=5; pedometer n=1 |
| Turner,2018 | breast(n=20), colorectal(n=2), prostate(n=1) | Undergoing and post treatment | Theory**,** BCTs; combine supervised and home-based exercise, exclusively home-based, exclusively supervised | usual care or 'waiting list' control | accelerometers or heart rate monitoring; self-reported |
| Abdin,2019 | breast cancer including invasive carcinoma and in situ disease | Undergoing and post treatment | tailored programme, motivational weekly supervised | relaxation wait-list control, usual care, Supervised Standard PA, maintain existing PA practice. | Unclear |
| Grimmett,2019 | Breast(n=11), mixed(n=7), Colorectal(n=4), Prostate(n=2), Esophagogastric(n=1), Lung(n=1), Testicular(n=1) | Undergoing and post treatment | home-based/group supervised exercise classes, counselling and group discussions, peer-led telephone consultation and mailed feedback report, printed materials and pedometer | Waitlist control, Usual care, Control group – asked not to change exercise behaviour for the entire follow-up period, Contact control - Cancer survivorship tip sheets (not PA related). Telephone calls as per intervention group to monitor symptoms | Actirgraph, n=4; Scot-PASQ, n=1; 7-DPARQ, n=5; GLTEQ, n=5; SQUASH, n=1; CHAMPS, n=1; Active Australia survey, n=1; IPAQ, n=1 |
| Schaffer,2019 | breast (n=5), colon or breast (n=2), prostate (n=1), acute leukemia (n=1), or others (n=3) | Undergoing and post treatment | supervised walking groups, educational sessions, self-directed training(exercise recommendations conveyed via phone with written materials/protocol, written printed materials, smartphone app, and DVD multimedia tool) | Usual care or non-exercise interventions | pedometers only, n=8; pedometers combined with a smartphone app (Smart After-Care; n=1), Wii Fit (Nintendo; n=1), or heart rate monitor (n=1); and a wireless sensor with triaxial accelerometer, gyroscope, and magnetometer (n=1)  GLTEQ, CHAMPS, Active Australia Survey, IPAQ-SF (in Korean) |
| Sheeran,2019 | mixed(n=44), breast(n=66), prostate(n=13), colorectal(n=6), other particular types of cancers (n=9) | Undergoing and post treatment | Prompt specific goal setting, Prompt self-monitoring of behavior, Prompt intention formation, and Prompt barrier identification | na | Unclear |
| Brunet,2020 | breast(n=7), mixed(n=3), prostate(n=1) | Undergoing and post treatment | In-person PA recommendation, tandardized PA-print materials and instructional yoga DVD, telephone-based PA counselling | Usual care, contact control, Standardized PA print materials | Modified Leisure Time Exercise Questionnaire, n = 5; IPAQ-SF, n = 1; 7-DPARQ, n = 7; Active Australia Survey, n = 1; CHAMPS, n = 1; Physical Activity Scale for the Elderly Saltin and Grimby Questionnaire, n = 1 |
| Dorri,2020 | BCS | post treatment (received treatment, including surgery, radiotherapy, or chemotherapy) | The web-based intervention, Fitbit + accelerometer, MijnAVL Portal, LoseIt, Map My Fitness + Actigraph GT3X + accelerometers, GAINFitness App, Smart After Care App + pedometer | No information or materials, Not mentioned, No intervention, Usual care waiting list control condition | accelerometers and pedometers, n = 4; PAM, n = 1; IPAQ, n = 3; GLTEQ7, n = 2; DPARQ, n = 1 |
| Mbous,2020 | adult CRC, inactive or not meeting ACS’ PA requirements at baseline (i.e., at least 150 min. of moderate intensity PA or 75 min. of vigorous intensity PA per week) | Undergoing and post treatment | Theory**,** BCTs | any parallel control group | 7-DPARQ, n = 3; GLTEQ, n = 2; CHAMPS, n = 1; IPAQ, n = 1; Total Physical Activity Questionnaire, n = 1; Physical Activity Scale for the Elderly, n = 1 |
| Blackwood,2021 | breast(n=3), mixed(n=1), Prostate(n=1), | Undergoing and post treatment | Actigraph GT3X+ accelerometer, Heart rate monitor + pedometer, smartphone apps, telephone calls + text messaging | no feedback or counseling during the exercise intervention | accelerometer, n = 1; pedometer, n = 1; Fitbit, n = 1; Active Australia Survey, n = 1; CHAMPS, n = 1; 7-DPARQ, n = 1; IPAQ-SF, n = 1; CHAMPS, n = 1 |
| Blount,2021 | BCS in full remission and had not received treatment for at least six months | Post treatment | Smartwatch + Facebook, Accelerometer + telephone, Garmin Vivofit 2 activity tracker, behavioral feedback, goal setting, telephone health coaching sessions | not have smartwatch, usual care, no interventions, | ActiGraph GT3X+, Polar M400.GT3X, ActivPAL, |
| Cheung,2021 | pediatric cancer had completed cancer treatment or on remission phase; aged 18 years or below | Post treatment | wearable technology with social media component, active video gaming, psycho-educational; Face-to-face, Web- and phone-based, | compares the intervention to an alternative intervention | accelerometer, n = 2; pedometer, n = 1; actiGraph GT3X+, n = 1; SenseWear Pro 2 Armband, n = 1; The Chinese University of Hong Kong: Physical Activity Rating for Children and Youth, n = 2; |
| Ester,2021 | breast(n=24), mixed(n=23), prostate(n=5) | Undergoing and post treatment | Theory, BCT, Website, text messaging, phone counseling, Print materials, in-person counseling, group interaction | unclear | Accelerometers, n = 26; pedometers, n = 7; GLTEQ, n = 16; IPAQ, n = 10; |
| Ibeggazene,2021 | breast(n=2), colorectal(n=1) | Undergoing and post treatment | home-based physical activity counselling with self-monitoring of activity via heart rate, pedometers and an exercise log with and weekly phone calls, Theory**,** BCTs | usual care | 7-DPARQ, n = 2; accelerometry, n = 2; CHAMPS, n = 1 |
| Khoo,2021 | breast(n=11), mixed(n=8), Colorectal(n=2), prostate(n=2), pediatric(n=1), Endometrial(n=1), Hepatocellular(n=1), Brain tumor(n=1) | Undergoing and post treatment | Activity tracker and text messages, set PA goals, behavioral and social support information | No intervention, Printed material, self-learning program for cancer survival and pedometer, Not applicable | Unclear;  MVPA(min/w），METs，activity trackers, step counts (Steps/d), vigorous PA (METs/w) |
| Liu,2021 | female BCS | Within three years of their last active treatment (not including hormone therapy) | phone calls, individual counselling sessions and group discussions, online/email delivery, material | usual care-not to change their physical activity level, or were provided with limited breast cancer and physical activity information;  contact control-a comparable frequency of contact but without the primary intervention components | pedometers, n = 7; the achievement of an activity level goal (ranging from 90 to 150 min a week ), the percentage of prescribed sessions completed |
| Meyer-Schwickerath,2021 | breast(n=4), mixed(n=5), Endometrial(n=3), Prostate(n=1), Colorectal(n=1) | Undergoing and post treatment | strategies to reduce stress, improve mood, pedometer, cognitive and behavioral PA skills/ Educational Support Program, Motivational Interviewing | usual care, Wait-list control, participants received standard information, received print materials containing PA guidelines | 7-DPARQ, n =5; CHAMPS, n = 1; Godin Leisure score index, n = 3; FFKA, n = 1; interview-administered physical activity questionnaire, n = 1; pedometer, n =2 |
| Pudkasam,2021 | female BCS | Completed primary treatments at least three months(continuing hormonal and immune targeted therapy) | Motivation strategies, Pedometer, counselling based on Theory Model, Print materials | Wait listed control, Pedometer + Standard PA guideline, Pedometer + Standard PA guideline, Maintain usual activity. | Unclear;  S-MVPA (min/ week), Daily steps, O-MVPA (min/week) |
| Hailey,2022 | female BCS | Completed treatment(except hormonal therapy within the past 5 years) | Theory, emails, face to face | usual care or ‘waiting list’ control | Unclear;  MET-hours/ week, MET-min/week, MVPA min/ day, 8-min walk test, MPA min |
| Singh,2022 | breast cancer(n=15), mixed cancer(n=13), prostate cancer(n=3), colorectal cancer(n=3), leukaemia(n=1) | Undergoing and post treatment | Theory, wearable physical activity trackers, counselling or instruction session, phone or email-based | usual care | Pedometer, n = 25; 7 trials (20%) involved Fitbit-based, n = 7; Polar, n = 2; Garmin, n = 1 |
